# Revealing the true face behind the mask of ALK-positive anaplastic large cell lymphoma (ALCL)

**DOI:** 10.1007/s00277-020-04247-4

**Published:** 2020-09-08

**Authors:** Hannah Eisfeld, Stefan Kircher, Andreas Rosenwald, Ioannis Anagnostopoulos, Mathias Werner, Nikolaus Gaßler, Gunter Wolf, Lukas Lehmkuhl, Ulf Teichgräber, Falk Gühne, Andreas Darr, Martin Freesmeyer, Wolfram Weschenfelder, Gunther Hofmann, Ramazan Dalkilic, Rolf Kalff, Andreas Hochhaus, Karin G. Schrenk

**Affiliations:** 1 Abteilung Hämatologie und Internistische Onkologie, Klinik für Innere Medizin II, Universitätsklinikum Jena, Am Klinikum 1, 07747 Jena, Germany; 2 Pathologisches Institut, Universität Würzburg, Josef-Schneider-Straße 2, 97080 Würzburg, Germany; 3 Institut für Pathologie, Vivantes Medizinisches Versorgungszentrum Berlin, Fröbelstraße 15, 10405 Berlin, Germany; 4 Institut für Rechtsmedizin, Sektion Pathologie, Universitätsklinikum Jena, Am Klinikum 1, 07747 Jena, Germany; 5 Abteilung Nephrologie, Rheumatologie/Osteologie und Endokrinologie/Stoffwechselerkrankungen, Klinik für Innere Medizin III, Universitätsklinikum Jena, Am Klinikum 1, 07747 Jena, Germany; 6 Klinik für Diagnostische Radiologie, Rhön-Klinikum Campus Bad Neustadt, Von-Guttenberg-Str. 11, 97616 Bad Neustadt a. d. Saale, Germany; 7 Institut für Diagnostische und Interventionelle Radiologie, Universitätsklinikum Jena, Am Klinikum 1, 07747 Jena, Germany; 8 Klinik für Nuklearmedizin, Universitätsklinikum Jena, Am Klinikum 1, 07747 Jena, Germany; 9 Klinik für Unfall-, Hand- und Wiederherstellungschirurgie, Universitätsklinikum Jena, Am Klinikum 1, 07747 Jena, Germany; 10 Klinik für Neurochirurgie, Universitätsklinikum Jena, Am Klinikum 1, 07747 Jena, Germany

Dear Editor,

Anaplastic large cell lymphoma (ALCL) is the third most common peripheral T cell lymphoma [1] and is divided into anaplastic lymphoma receptor kinase (ALK)-positive and ALK-negative ALCL [2]. In 80% of ALK-positive ALCL, ALK is constitutively activated as a result of nucleophosmin (NPM)-ALK translocation. ALK belongs to the insulin receptor superfamily [3, 4]. Whereas the 5-year overall survival in ALK-positive ALCL is 70%, patients with ALK-negative ALCL have a 5-year survival of 32–50% [1, 5, 6]. On histology, ALCL shows large pleomorphic neoplastic cells with often horseshoe-shaped nuclei and strong expression of CD30, a cytokine receptor from the tumor necrosis factor receptor family [7, 8]. However, besides these characteristic findings, there is a broad range of morphological variations [8, 9].

**Fig. 1:**
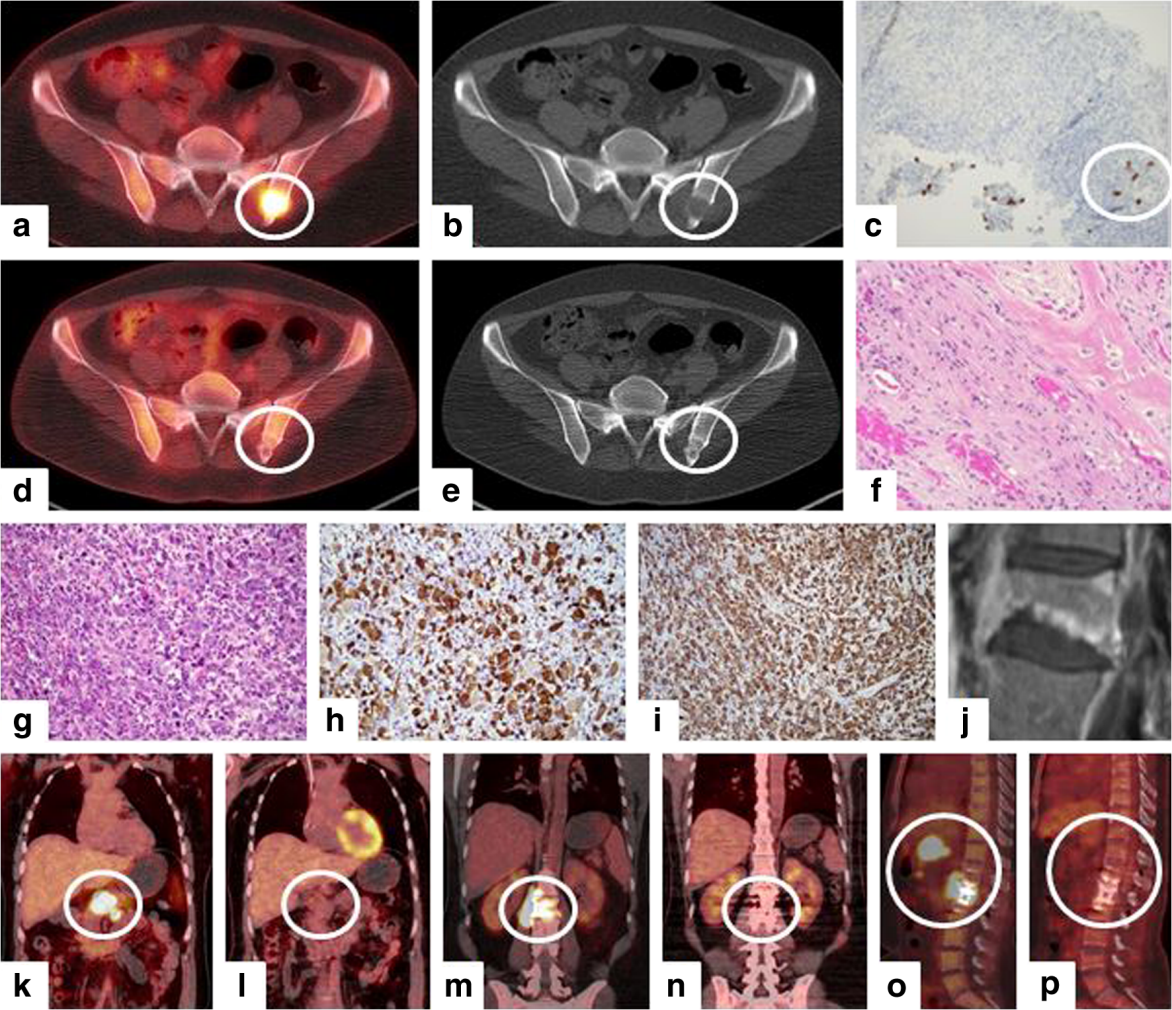
Upper and middle panel: initial manifestations of ALK-positive ALCL. **a** PET and **b** CT imaging demonstrating hypermetabolic bone lesions in the left iliac bone. Initial histology with **c** few CD30-positive cells (× 200 magnification) and **f** metaplastic woven bone as well as chronic inflammatory infiltration (× 20 magnification), consistent with the diagnosis of chronic recurrent multifocal osteomyelitis (CRMO). **d** PET and **e** CT scan after therapy with steroids and infliximab. All initial hypermetabolic bone lesions had disappeared. **j** MRI scan of fractured L2. Histology revealed the diagnosis of ALK-positive anaplastic large cell lymphoma. **g** Hematoxylin staining (× 400 magnification), **h** ALK staining (× 400 magnification), and **i** CD30 staining (× 200 magnification). Lower panel: PET imaging before and after treatment with chemotherapy. New hypermetabolic lesions were found **k** in the pancreas and **m**, **o** paravertebral in the region of the resected L2 vertebral body with infiltration into the psoas muscle. **l**, **n**, **p** After treatment with 6 cycles of combination chemotherapy according to the CHOEP protocol complete remission was achieved (Deauville-5P-response score 1)

We present an unusual case of a 38-year-old female patient with challenging diagnostic workup of an ALK-positive ALCL due to the variable histology and clinical course of this lymphoma. The patient initially complained of lower back pain and fever. FDG-positron emission tomography and computerized tomography (PET-CT) of the thorax and abdomen revealed multiple bone lesions (Fig. 1a, b). The patient had an unremarkable medical and family history and no occurrence of B-symptoms. Clinical examination showed no abnormalities. All other examinations including bronchoscopy, gynecologic assessment, gastroscopy, colonoscopy, and abdominal ultrasound did not reveal any abnormalities. The laboratory results were within normal limits except elevated C-reactive protein (CRP) levels with 56.9 mg/l (normal range < 7.5 mg/l). A CT-guided core needle biopsy of an osteolytic lesion of the left iliac bone was performed. The histopathologic results showed an infectious or inflammatory process. No malignancies could be diagnosed; however, CD30-positive cells were found at low frequency, which could not be further evaluated due to the scarcity of the available tissue (Fig. 1c). A second bone biopsy of this lesion revealed metaplastic woven bone with chronic inflammatory infiltration, consistent with chronic recurrent multifocal osteomyelitis (CRMO) (Fig. 1f). Bisphosphonates and corticosteroid therapy was commenced. Because of refractory pain, tumor necrosis alpha inhibitor infliximab was given. Magnetic resonance imaging (MRI) scan revealed a L2 fracture (Fig. 1j). Therefore, dorsal stabilizing surgery was performed. Unexpectedly, the bone histology revealed an ALK-positive ALCL (Fig. 1g–i). Seven months after her first visit, the diagnosis was finally made. The patient reported loss of weight over the last 14 months of 18 kg of body weight. She was still suffering from pain in the hip and the lumbar spine and had upper abdominal spasms. A PET-CT was performed, revealing hypermetabolic paravertebral lesions in the region of the resected L2 with infiltration of the psoas muscle (Fig. 1m, o) and in the pancreas (Fig. 1k). However, all the previously known osteolytic lesions had disappeared (Fig. 1d, e). After pre-phase chemotherapy, the patient received six cycles of bi-weekly CHOEP (cyclophosphamide, doxorubicin, vincristine, etoposide, and prednisone). After the sixth cycle, PET-CT showed a complete response, Deauville-5P-response score 1 (Fig. 1l, n, p). The patient has been in complete remission for 33 months.

Since the discovery of ALCL by Stein et al. [10] due to the characteristic staining with the anti-Ki-1-antibody labeling CD30, high variability in the morphologic appearance of ALCL has been recognized [9, 10]. The major histological classification includes the common type, the small cell type, and the lymphohistiocytic variant of ALCL. Other variants resemble Hodgkin’s disease with giant cells, signet-ring cell tumors, sarcoidosis, sarcoma, or inflammation with a high amount of neutrophil and eosinophil granulocytes [9]. Moreover, approximately 30% of ALK-positive ALCLs have a mixture of several histological variants [9].

This case shows the difficulties in diagnosing ALK-positive ALCL due to the variable clinical course including bone infiltration. A major challenge is a histological diagnosis since anaplastic large cell lymphoma may present with heterogeneous morphology, expansion of inflammatory cells, and few CD30-positive cells, especially if limited biopsy material is warranted, contributing to delayed diagnosis. In patients with bone lesions and unspecific histological result, ALCL should be considered with high priority in differential diagnosis.
